# A lightweight Chinese–English translation model integrating compressed BERT attention and phrase discard mechanism

**DOI:** 10.3389/fnbot.2026.1795490

**Published:** 2026-05-08

**Authors:** Xin Qi, Hang Bao

**Affiliations:** Liaoning National Normal College, Shenyang, China

**Keywords:** BERT knowledge enhancement, Chinese–English machine translation, graph convolutional network, learnable compression vector, phrase discard mechanism

## Abstract

Chinese–English machine translation based on neural network model strictly adopts the sequential modeling method of encoder–decoder. However, this traditional method cannot make effective use of syntactic information and linguistic hierarchy information. Therefore, to integrate syntactic structure information into Chinese–English machine translation to improve its translation performance, this paper proposes a new Chinese–English machine translation method based on graph convolutional network and BERT (Bidirectional Encoder Representation from Transformers) knowledge enhancement. In this work, we present an enhanced approach to neural machine translation that integrates multiple techniques to improve translation quality. The multi-BERT context is first compressed and aligned into the semantic space of the translation model using learnable compression vectors. This alignment ensures that the rich contextual information from BERT is effectively utilized within our translation framework. At the end of source language, we employ a dual encoder to encode both the source sentence and its syntactic dependency tree, thereby capturing both lexical and structural information. To further enrich the source-side semantic representation, the compression vector is concatenated with the input vector of the encoder. Additionally, we introduce a phrase discard mechanism that randomly discards target phrases during training. This mechanism enhances the model’s robustness against mistranslated phrases, thereby reducing their impact on subsequent phrase translations. Experiments on NIST dataset demonstrate the effectiveness of our proposed lightweight Chinese–English translation method. Different from general-purpose large chatbot models (e.g., ChatGPT) with high computing costs, this model achieves 39.68 BLEU with low parameters, solving issues of low-resource scenarios and phrase mistranslation. It offers a novel lightweight paradigm for private-oriented translation chatbots, outperforming the baseline Transformer (35.75 BLEU) significantly.

## 1 Introduction

Words or characters can be thought of as the basic units that make up language, however, people often use phrases to express specific meanings. For example, considering the English translation “China and Serbia have very good relations.”, the sentence becomes easier to understand if it is split into: “[China and Serbia] [have] [very good] [relations].”, where each bracketing word forms a phrase. If these phrases are not translated accurately, it will greatly affect the quality of the translation (Maharani et al., 2023; Tektigul et al., 2023).

Since the traditional neural network model assigns the same weight to each word in training, it does not take into account the different importance of each word (Bowers et al., 2023), therefore, a method is generated by considering some additional information as the target end word. The traditional neural network model assigns different training weights to each word in training, which is called adaptive training. Li et al. (2023) believed that low-frequency words were difficult to train and should have higher training weights. Therefore, two heuristic objective functions were proposed to assign different weights to target words with different frequencies. For Exponential targets, the weight monotonically increased with decreasing frequency; For Chi-Square goals, only words that were meaningful but relatively low-frequency could be assigned greater weight. Vitta et al. (2023) believed that words with polysemy should have a higher tolerance in training, and the training weight of polysemy words should be appropriately reduced. Therefore, the training target BMI (bilingual mutual information) of word level was proposed, the learning difficulty of each word was measured from the bilingual perspective, and the word weight was dynamically adjusted. Previous studies only used word-level auxiliary information, while the method in this paper uses phrase-level auxiliary information, in order to restrict the model to remember phrases by changing the weight.

Traditional phrase-based statistical machine translation (SMT) methods have been shown to be superior to word-based methods (Durrani et al., 2015). However, works on phrases in modern neural machine translation (NMT) methods (Yu et al., 2024; Wang et al., 2017) have focused on using phrases generated by external tools to provide additional information to neural network models. For example, Devi et al. (2023) used phrases generated by the SMT model to extend beam searching. Razaq et al. (2023) used SWAN (Sleep–WAke Networks) method to obtain phrase structure and model it. However, with the exception of external phrase information, the model could not accurately translate phrases in the training set. In the WMT (Workshop on Statistical Machine Translation) 14 Anglo-German data set, the standard NMT model had a translation accuracy of only 27.5% for 4-word phrases in the training set, indicating that a large number of phrases were not translated accurately. Because the NMT model minimized the loss of each word, this could result in no explicit constraints on memorizing phrases, so even phrases in the training set could be mistranslated. Li (2024) introduced an attention-based hybrid search algorithm, which extended the beam search of NMT through SMT phrase translation. Moe (2023) translated phrases by integrating the target phrase from the phrase memory into the NMT, where the phrase memory was provided by the SMT model, and the NMT decoder then selected a phrase from the phrase memory or a word from the word with the highest probability to generate it. Banik (2023) proposed to use SWAN to model the phrase structure in the target language. The new method in this paper takes advantage of the nature of the model itself to find phrases and improve translation quality without additional parameters or information.

The model is regularized to memorize phrases using adaptive training based on word-level, which encourages the model to focus on the translation of specific words by assigning different weights to each word. For example, since the standard Transformer (Vaswani et al., 2017) had low translation accuracy for low-frequency words, the constraint model (Liu et al., 2022) focused on low-frequency and meaningful words, which alleviated the problem that the model over-fitted high-frequency words while ignoring those low-frequency words. Xu et al. (2021) aimed to use Bilingual Mutual Information (BMI) to measure the learning difficulty of words, assigning more weight to words that were easy to learn and less weight to words that were not easy to learn.

The use of pre-trained language model (PLM) for transfer learning is a method that has received more attention. Pre-trained models such as GPT, BERT (Koroteev, 2021), RoBERTa, XLM and XLNET are trained on massive monolingual data and learn rich semantic representations. By migrating these pre-trained models to machine translation tasks, the representation and generalization performance of the translation models can be enhanced, thus improving the effect of translation in low-resource scenarios. Studies have shown that using PLM can achieve significant results in enhancing the performance of NMT models. Also some new models have been presented such as Cascade chaotic neural network (CCNN) (Abbasi et al., 2022; Abbasi, 2024). These methods are mainly divided into two categories: first, the parameters of PLM are fine-tuned as the initial parameters of downstream tasks; the second is to freeze the PLM and integrate the context embed it generates into the NMT model. However, fine-tuning PLM directly can affect the performance of NMT models by causing them to forget previously learned knowledge when training new tasks, a phenomenon known as “catastrophic forgetting.” Context embedding into PLM approaches are widely studied and effective methods (Nzetchou et al., 2022; Zhou et al., 2025), but these fusion methods are more complex and greatly increase the number of training parameters, resulting in a significant increase in the computational cost of training and reasoning processes. Furthermore, due to differences between PLM and NMT models in terms of pre-training tasks, model architecture, and training data domains, directly freezing and integrating PLM contextual embedding limits its potential in NMT models, limiting performance gains.

We specifically select the multilingual BERT (mBERT) rather than monolingual BERT variants (e.g., Chinese BERT or English BERT) for three compelling reasons in the context of Chinese–English translation. First, mBERT is jointly pre-trained on 104 languages, which helps capture semantic representations useful for Chinese–English translation that are crucial for translation tasks. In contrast, monolingual BERT models are trained independently on single-language corpora, lacking explicit cross-lingual supervision that could bridge the semantic gap between source and target languages. Second, for low-resource language pairs such as Chinese–English with limited parallel data, mBERT’s cross-lingual transfer capability allows knowledge transfer from high-resource languages during pre-training, providing richer contextual representations than monolingual counterparts. Third, using mBERT eliminates the need to maintain separate encoders for source and target languages, reducing model complexity and enabling unified representation learning. While monolingual BERT may offer stronger language-specific representations, empirical evidence in Table 1 demonstrates that our CAM effectively compresses mBERT’s cross-lingual knowledge, achieving superior translation performance (BLEU 35.60) compared to direct integration approaches. This validates that mBERT’s multilingual nature, combined with proper compression mechanisms, is particularly advantageous for neural machine translation scenarios.”

**Table 1:** Impact of different BERT models on translation quality.

Model	BLEU
Transformer	35.75
mBERT(enc-in)	34.37
XLM-R(enc-in)	35.49
BERT-Fused	36.22
mBERT-CAM(-DGT)	35.60
BERT-CAM(-DGT)	37.51^*^ (+2.76)

^*^Indicates a statistically significant improvement in BLEU score compared to the baseline system (Bootstrap resampling test, *n* = 2,695 [test set samples], 25 independent runs, *p* = 0.05).

This paper summarizes its contributions into three consistent aspects.

(1) We propose a novel lightweight Chinese–English translation framework integrating graph convolutional network and BERT knowledge enhancement to improve translation accuracy and efficiency for Chinese–English translation.
(2) We design a compressed attention module (CAM) to integrate multilingual BERT context embeddings into Transformer with linear complexity, avoiding catastrophic forgetting and excessive computational overhead. We also introduce a phrase discard mechanism (PDM) to enhance phrase-level translation robustness and a dual multi-granularity training (DGT) strategy to fully exploit bilingual knowledge, forming a complete lightweight training and inference pipeline.
(3) Extensive experiments on the NIST Chinese–English translation dataset show that our model achieves 39.68 BLEU, outperforming the baseline Transformer by 3.93 BLEU and surpassing other advanced methods. Further analyses including ablation studies, parameter efficiency evaluation, hyperparameter sensitivity testing, and qualitative case studies consistently validate the effectiveness, robustness, and efficiency of each component.

## 2 Proposed Chinese–English machine translation model

First, we will introduce some basic concepts that are necessary for this article.

Neural machine translation (NMT) based on the encoder–decoder framework has become the mainstream paradigm for automatic translation. Early NMT systems relied on recurrent neural networks (RNNs) for sequence modeling, while later Transformer models fully adopted self-attention and achieved state-of-the-art performance by modeling long-range dependencies. Most conventional NMT methods focus on high-resource language pairs with abundant parallel data, but yields unsatisfactory performance in low-resource settings due to insufficient supervision and weak representation ability. In contrast, this work targets lightweight and efficient Chinese–English translation by integrating syntactic structure and pre-trained language model knowledge. Machine translation is the process of using computers to convert one language into another. Given a source language sentence $X=\{x_{1},x_{2},\cdots ,x_{i},\cdots ,x_{N}\}$ with N words and a sentence $Y=\{y_{1},y_{2},\cdots ,y_{i},\cdots ,y_{M}\}$ in the target language with M words, NMT is decompose sentence-level probabilities into the product of word-level probabilities as shown in Equation 1:


$$P[y|x;\theta ]=\prod_{j=1}^{N}P[y_{j}|x|,y_{j};\theta ]$$ (1)

Where θ represents the conversion formula between the source language and the target language.

**Figure 1:**
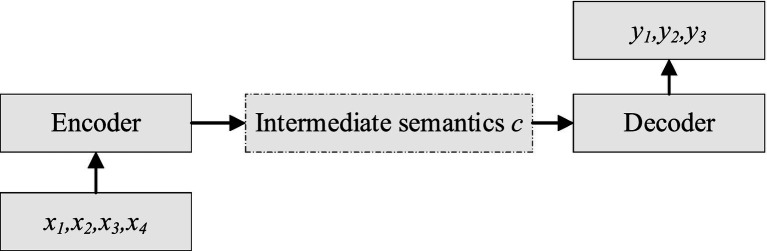
Encoder–decoder model.

A neural machine translation model usually consists of an encoder, a decoder, and some methods of constructing the connection between the encoder and decoder. For example, in the attention mechanism, the encoder receives a sequence of source language sentences, converts it into an intermediate representation, and the decoder generates a translation of this intermediate representation, as shown in Figure 1.

Low-resource NMT aims to improve translation quality with limited parallel data. Recent methods can be categorized into three lines: data augmentation, transfer learning, and architectural enhancement. Data augmentation techniques include back-translation, token-level shuffling, and multi-granularity sample expansion (Hassan and Mahmood, 2018; Fu et al., 2019; Chen et al., 2017). Transfer learning approaches leverage monolingual corpora or cross-lingual signals to initialize or regularize model training (van der Veen, 2023). Architectural methods enhance the encoder with external linguistic knowledge such as syntax, part-of-speech tags, or dependency trees to reduce data dependence (Dai et al., 2024; Das et al., 2023; Jiang and Yin, 2023). However, most existing methods either ignore syntactic inductive bias or introduce heavy computation when integrating pre-trained models. This work addresses both issues via a lightweight compression mechanism and syntactic fusion strategy.

Pre-trained language models (PLMs) such as BERT, mBERT, XLM-R, and XLNet have been widely used to improve NMT by providing rich contextual representations (Glorot et al., 2011; Ott, 2019). Two mainstream integration strategies exist: fine-tuning PLM parameters directly, or freezing PLMs and fusing their embeddings into NMT. However, direct fine-tuning causes catastrophic forgetting, while naive fusion significantly increases computational complexity and model size. To address these problems, some lightweight adaptation methods have been proposed, including knowledge distillation, low-rank decomposition, and feature compression. Nevertheless, few methods simultaneously achieve cross-lingual alignment, syntactic fusion, linear complexity, and phrase-level robustness.

Phrase-level modeling has long been proven effective for translation. Early phrase-based statistical methods were later integrated into NMT via external phrase tables or memory mechanisms. Recent studies use phrase-aware attention or phrase-level training objectives to encourage the model to memorize frequent phrases. Meanwhile, syntactic information such as dependency trees has been incorporated via graph convolutional networks (GCN) to improve structure awareness. Despite these advances, phrase robustness and syntactic encoding are rarely combined with lightweight cross-lingual knowledge enhancement. This work unifies phrase-level training, syntactic GCN encoding, and compressed BERT fusion within a single lightweight framework.

Neural machine translation is a sequence-to-sequence task. The goal is to establish a mapping relationship from sentence $x=(x_{1},x_{2},\cdots ,x_{n})$ in the source language to sentence $y=(y_{1},y_{2},\cdots ,y_{m})$ in the target language. The current mainstream NMT models all adopt the encoder–decoder architecture (Zhou et al., 2023; Khan, 2024).

In this architecture, the source language words are first converted by the encoder to a hidden representation Z , as in Equation 2. Then, the decoder uses Z and the previously generated target word sequence $y=(y_{1},y_{2},\cdots ,y_{t-1})$ as input to generate the representation $h_{t}$ of the t-th target word, i.e., Equation 3:


$$Z=\text{Encoder}(x)=(z_{1},z_{2},\cdots ,z_{n})$$ (2)

$$h_{t}=\text{Decoder}(Z,y)$$ (3)

Then, $h_{t}$ is mapped to the dimension ∣V∣ of the target vocabulary using a linear projection, and the probability distribution for generating word $y_{t}$ at time t is calculated using the Softmax function, as shown in Equation 4. Finally, the word with the largest probability value is taken as the target word $y_{t}$ for the prediction at time t . The probability of the whole sentence is expressed as the joint probability distribution of each word in the target language, as shown in Equation 5.


$$p_{q}(y_{t}|Z,y;q)=\text{Softmax}(\text{Linear}(h_{t}))$$ (4)

$$p_{q}(y|x|;\vartheta )=\sum_{t=1}^{m}p_{q}(y_{t}|Z,y;q)$$ (5)

Where ϑ represents the parameter of the neural network, which can be optimized by minimizing the log-likelihood between the predicted and true distribution of the target language, as shown in Equation 6.


$$\mathrm{Los}s_{\mathrm{nll}}=-\sum_{(x,y)\in D}\log p_{q}(y|x;\vartheta )$$ (6)

Where $\mathrm{Los}s_{\mathrm{nll}}$ is negative log-likelihood loss. (x,y) is source-target sentence pair. x is source language sentence. y is target language reference sentence. D is parallel training dataset. $p_{q}$ is model’s conditional probability.

### 2.1 Overall architecture

**Figure 2:**
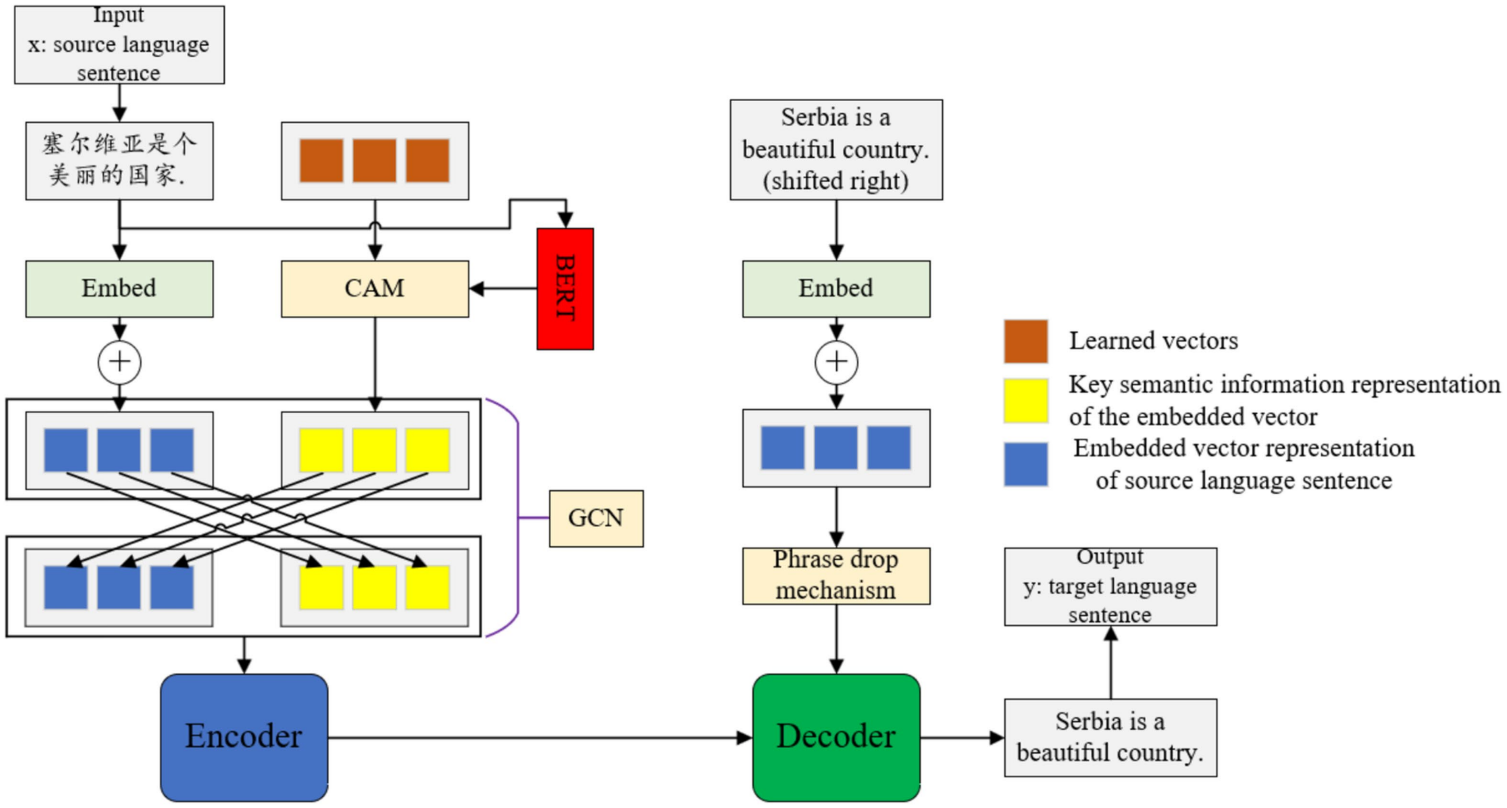
The architecture of proposed Chinese–English machine translation model.

The overall proposed Chinese–English machine translation model architecture in this paper is shown in Figure 2. The model is mainly composed of four modules: mBERT, CAM, encoder and decoder. CAM is used to compress the output representation of mBERT and splice it with the input representation of the encoder to incorporate richer contextual information. For any given source language sequence $x=(x_{1},x_{2},L,x_{n})$ to target language sentence $y=(y_{1},y_{2},L,y_{m})$ , the flowchart of the proposed method is as follows.

1) Encoder input.

Given the source language sentence x , x is transformed into a vector representation by embedding the word layer, and then positional encoding is added to give positional information to each word element, thus obtaining the encoder input E , as shown in Equation 7:


E=Embed(x)+PE(x) (7)

where, Embed represents the word embedding layer, PE represents the location encoding layer, $E\in R^{n\times d_{\text{model}}}$ . n is the source language sequence length encoded by the NMT model. $d_{\text{model}}$ is the embedded dimension of the NMT model.

2) mBERT semantic compression.

In order to capture the richer semantic information of sentence x in the source language, we use mBERT to obtain its context embedding B . Meanwhile, we use a learnable set of lightweight continuous vectors C as the backbone sequence for information extraction. Through the cross-attention layer of CAM, we integrate the key semantic information in B into C to generate a new compressed representation $\tilde{C}$ . This process can be expressed by the following Equation 8,9:


B=mBERT(x) (8)

$$\tilde{C}=\mathrm{CAM}(C,B)$$ (9)

where, $B\in R^{l\times d}$ , l is the length of the source language sequence encoded by mBERT. d is the embedding dimension of mBERT. C and $\tilde{C}$ have the same dimension and are both $R^{k\times d_{\text{model}}}$ . k is the length of the compression vector, and it is much less than l , which realizes the effective extraction and compression of the original information B .

3) Representation fusion.

After getting the compressed vector $\tilde{C}$ of the CAM output, we simply concatenate it with the encoder input E to get the enhanced source language representation $\tilde{E}$ . This process can be expressed as shown in Equation 10:


$$\tilde{E}=E\|\tilde{C}=(e_{1},e_{2},\cdots ,e_{n},c_{1},\cdots ,c_{k})$$ (10)

where ‖ represents the concatenation operation in the sequence dimension, $\tilde{E}\in R^{(n+k)\times d_{\text{model}}}$ . In this way, the source language representation of NMT can focus on the mBERT compressed sequence $\tilde{C}$ as a suffix in the attention mechanism, thus enhancing the representation capability of the source side.

4) Standard NMT process.

We use an enhanced source language to represent $\tilde{E}$ , which is fed into the Transformer encoder. The encoder obtains the semantic representation of the source input sequence through stacked self-attention layers and feed-forward networks, as shown in Equation 6. In the decoding stage, the decoder combines the generated target language sequence and the encoder output to gradually produce the target language representation, as shown in Equation 7. Finally, the decoder output is converted into a probability distribution on the vocabulary of the target language through the linear layer and the Softmax function to generate the translation result, as shown in Equation 8. The encoder and decoder of the proposed method are the same as the standard Transformer architecture, but by introducing CAM to compress mBERT knowledge, richer semantic information can be injected into the NMT model, thus improving the translation performance succinct and effective.

#### 2.1.1 GCN-transformer-CAM fusion mechanism

To integrate syntactic structural information (from GCN) and compressed cross-lingual semantic information (from CAM) into the Transformer encoder in a dimensionally consistent and layer-wise collaborative manner, we design a dual-stream feature fusion pipeline with dimensional alignment and hierarchical interaction. The fusion strictly follows the principle of syntactic-semantic feature co-encoding and is implemented at the bottom encoder layer (Layer 1) of the Transformer (before self-attention computation) and intermediate encoder layer (Layer 3) (after feed-forward network computation), to ensure syntactic structure information is fully propagated and fused with semantic information throughout the encoding process.

The GCN takes the encoder input embedding E and the source language syntactic dependency tree as inputs, and outputs the syntactic structure representation $G\in {\mathbb{R}}^{n\times d_{\text{model}}}$ through the directed graph convolution operation. The dimensionality of G is strictly aligned with the Transformer model dimension $d_{\text{model}}$ and the CAM compressed vector $\tilde{C}\in {\mathbb{R}}^{k\times d_{\text{model}}}$ via a linear projection layer with weight matrix $W_{G}\in {\mathbb{R}}^{d_{\mathrm{gcn}}\times d_{\text{model}}}$ and bias $b_{G}\in {\mathbb{R}}^{d_{\text{model}}}$ if the raw GCN output dimension $d_{\mathrm{gcn}}$ is inconsistent with $d_{\text{model}}$ The process is shown in Equation 11:


$$G=\text{Linear}(G_{\mathrm{raw}})=G_{\mathrm{raw}}\cdot W_{G}+b_{G}$$ (11)

Where $G_{\mathrm{raw}}$ is the original output of the GCN for the syntactic dependency tree, and the linear projection layer is jointly trained with the entire model to learn syntactic feature mapping adapted to Chinese–English translation.

At the first layer of the Transformer encoder (the bottom layer, prior to self-attention), we perform concatenation + element-wise addition to fuse the GCN syntactic representation G , the original encoder input E , and the CAM compressed semantic vector $\tilde{C}$ . This fusion step injects both syntactic and compressed BERT semantic information into the Transformer at the initial encoding stage, and the fused feature $F_{1}\in {\mathbb{R}}^{(n+k)\times d_{\text{model}}}$ is defined as shown in Equations 12,13:


$$\hat{E}=E+G$$ (12)

$$F_{1}=\hat{E}\|\tilde{C}$$ (13)

Where $\hat{E}$ implements element-wise addition of syntactic features G and the original lexical-positional embedding E (dimensionality $n\times d_{\text{model}}$ ) to realize lexical-syntactic fusion, and $F_{1}$ splices the CAM compressed semantic vector $\tilde{C}$ (dimensionality $k\times d_{\text{model}}$ ) in the sequence dimension, generating the final input feature $F_{1}$ for the Transformer encoder Layer 1. $F_{1}$ is directly fed into the self-attention layer of Layer 1 as the encoder’s enhanced input.

At the third layer of the Transformer encoder (the intermediate layer, after the feed-forward network (FFN) of Layer 3), we introduce a GCN residual fusion mechanism to reinforce syntactic structure information, which prevents the attenuation of syntactic features in deep Transformer layers and realizes the interaction between GCN outputs and the CAM-semantic features that have undergone multi-layer self-attention encoding. Let the output of the FFN in Transformer encoder Layer 3 be $F_{3-\mathrm{FFN}}\in {\mathbb{R}}^{(n+k)\times d_{\text{model}}}$ ; we first perform a sequence padding on the GCN syntactic representation G (from $n\times d_{\text{model}}$ to $(n+k)\times d_{\text{model}}$ ) by zero-padding the last k positions (consistent with the CAM vector’s sequence length), generating the padded GCN representation $\hat{G}\in {\mathbb{R}}^{(n+k)\times d_{\text{model}}}$ . The fused feature $F_{3}\in {\mathbb{R}}^{(n+k)\times d_{\text{model}}}$ for Layer 3 is then defined as shown in Equation 14:


$$F_{3}=F_{3-\mathrm{FFN}}+\alpha \cdot \hat{G}$$ (14)

Where α∈(0,1] is a learnable fusion weight (initialized to 0.5) that dynamically adjusts the contribution of syntactic features in the intermediate encoding stage. $F_{3}$ is used as the input of the Layer 4 self-attention layer, and the learnable weight α is optimized with the model to balance syntactic and semantic feature contributions for different sentence structures (e.g., complex long sentences vs. simple short sentences).

After Layer 3 fusion, the fused features $F_{3}$ propagate through the remaining Transformer encoder layers (Layer 4 to Layer 6) without additional GCN/CAM fusion operations. The final encoder output Z is thus a unified syntactic-semantic representation that integrates lexical information, positional information, compressed mBERT cross-lingual semantic information (CAM), and source language syntactic dependency tree information (GCN). This representation is fed into the Transformer decoder to generate the target language translation, ensuring both semantic and syntactic accuracy in translation.

### 2.2 Compressed attention module (CAM)

#### 2.2.1 CAM design

The Compressed Attention Module (CAM) is proposed to address the core pain points of integrating pre-trained language models (PLMs) into Neural Machine Translation (NMT), high computational complexity, catastrophic forgetting, and semantic misalignment between PLM and NMT semantic spaces. It differs fundamentally from mainstream BERT compression/adaptation methods such as DistilBERT (knowledge distillation) and XLM-R (cross-lingual pre-training and direct embedding integration), as well as naive BERT fusion methods (e.g., BERT-Fused).

All design choices of CAM are driven by the practical challenges of PLM integration into low-resource Chinese–English NMT, and each choice is verified by experimental results in this paper:

(1) Frozen PLM + learnable lightweight compression vectors ( $C\in R^{k\times d_{\text{model}}}$ ). Freezing mBERT/XLM-R avoids catastrophic forgetting of pre-trained cross-lingual knowledge (a critical issue for fine-tuning-based PLM integration), while the learnable vectors enable task-specific optimization for Chinese–English translation without massive parameter addition.
(2) Fixed-length compression ( k≪l ). The length of compression vectors is independent of the input sequence length l , which ensures the model can efficiently process long sentences and avoid noise accumulation from overlong PLM embeddings.
(3) Self-attention + cross-attention dual layers. Self-attention enables internal interaction of compression vectors to capture global semantic correlations, and cross-attention merges frozen PLM embeddings into the compression vector space to achieve accurate semantic alignment.
(4) Sequence-dimension concatenation with NMT encoder input. Splicing the compressed PLM vector $\hat{C}$ with the NMT encoder input E makes the NMT’s attention mechanism focus on PLM’s key semantic information as a suffix, enhancing the source language representation ability without changing the original Transformer encoder/decoder architecture.

The proposed CAM is designed to effectively extract the core semantic information in mBERT context embedding with a set of fixed-length vectors. Since the input text length l is variable, we introduce the learnable lightweight compression vector $C\in R^{k\times d_{\text{model}}}$ as a fixed input to CAM. In CAM, we use a self-attention layer to let the vectors in C interact internally, and a cross-attention layer to let C merge with the frozen mBERT representation (Go et al., 2023; Ma et al., 2023).

**Figure 3:**
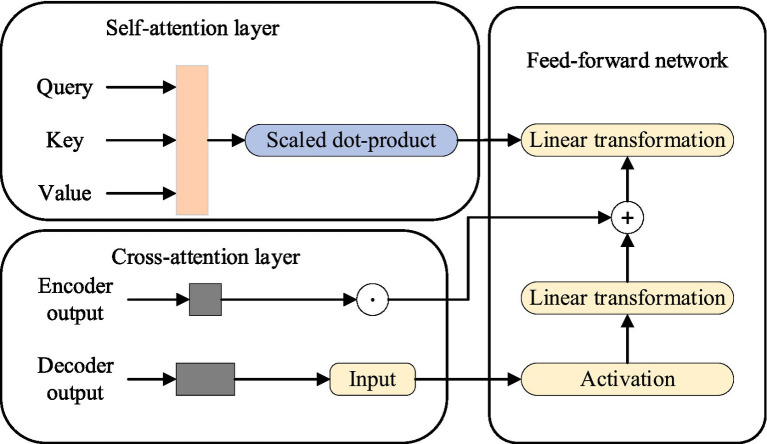
CAM structure.

The length k of the compressed vector C is independent of the length of the input text sequence l , and k≪l . This means that regardless of the length of the input sequence, we can use a fixed-length *C* to represent its key semantics and provide richer contextual information for merging with other embeddings by concatenating them in the timing. In this way, our model is able to process long text sequences more efficiently while accurately capturing key semantic information. Specifically, CAM consists of three parts: self-attention layer, cross-attention layer, and feed-forward network layer, as shown in Figure 3.

1) Self-attention layer.

First, we initialize a set of lightweight learnable compression vectors, $C\in R^{k\times d_{\text{model}}}$ , as inputs to CAM, which fully interact through the self-attention layer. Specifically, we use three different parameter matrices $W_{Q}^{C}$ , $W_{K}^{C}$ , $W_{V}^{C}$ to project the compression vector C as a query vector $Q^{C}=(q_{1}^{C},\cdots ,q_{k}^{C})$ , a key vector $K^{C}=(k_{1}^{C},\cdots ,k_{k}^{C})$ , and a value vector $V^{C}=(v_{1}^{C},\cdots ,v_{k}^{C})$ .

Then, we calculate the attention weight $e_{\mathrm{ij}}$ of the i-th query vector $q_{i}\in Q^{C}$ and the j-th key vector $k_{j}\in K^{C}$ , and normalize it with Softmax function to get $a_{\mathrm{ij}}$ , as shown in Equation 15,16.


$$e_{\mathrm{ij}}=\frac{q_{i}\times k_{i}}{\sqrt{d_{\text{model}}}}$$ (15)

$$a_{\mathrm{ij}}=\text{Softmax}(e_{\mathrm{ij}})$$ (16)

Finally, the output $C^{\prime }$ of the self-attention module is calculated by following Equation 17,18:


$$C^{\prime }=\text{Self}-\mathrm{att}(Q^{C},K^{C},V^{C})=(c\prime_{1},\cdots ,c\prime_{k})$$ (17)

$$c\prime_{i}=\sum_{j=1}^{k}a_{\mathrm{ij}}\times v_{j}$$ (18)

where $v_{j}\in V^{C}$ represents the j-th value vector.

2) Cross attention layer.

In this layer, the compressed vector $C^{\prime }$ interacts with the frozen mBERT output context embedding $B\in R^{l\times d}$ . We use the parameter matrix $W_{Q}^{C\prime }=R^{d_{\text{model}}\times d_{\text{model}}}$ to convert $C^{\prime }$ into the query vector $Q^{C\prime }=(q_{1}^{C\prime },\cdots ,q_{k}^{C\prime })$ , and use $W_{K}^{B}$ , $W_{V}^{B}$ to project the embedded dimension d of mBERT onto the dimension $d_{\text{model}}$ of NMT, yielding the key vector $K^{B}=(k_{1}^{B},\cdots ,k_{l}^{B})$ and value vector $V^{B}=(v_{1}^{B},\cdots ,v_{l}^{B})$ of B . Then, it uses cross attention to embed the context B into $C^{\prime }$ , as shown in Equation 19:


$$C^{\prime \prime }=\text{Cross}-\mathrm{att}(Q^{C\prime },K^{B},V^{B})=(c\prime \prime_{1},\cdots ,c\prime \prime_{k})$$ (19)

3) Feed-forward network layer.

Each position of *C″* is transformed through a feed-forward network, consisting of two linear transformation layers and a ReLU (Rectified Linear Unit) activation function. Note that in deep learning practice, “ReLU” is commonly used as the standard term for this rectified linear activation function, following the widespread convention in the field. The calculation process is shown in Equation 20. Moreover, we use residual joins and layer normalization behind each sub-layer. Finally, The output vector $\tilde{C}$ , which compresses the contextual embedding B of the text sequence, it can enhance the encoder embedding representation of the NMT model by concatenating it.


$$\tilde{C}=\mathrm{FFN}(Q^{\prime \prime })=\max(0,C^{\prime \prime }\times W_{1}+b_{1})W_{2}+b_{2}$$ (20)

#### 2.2.2 Computational complexity of CAM

This subsection provides a formal analysis of the computational complexity (FLOPs, floating-point operations) for the Compressed Attention Module (CAM), including its three core sublayers: self-attention, cross-attention, and feed-forward network (FFN). We also prove the linear complexity of CAM for input sequence length l , which is the key design advantage over traditional BERT fusion methods ( $O(l^{2})$ complexity).

1) Self-attention layer of CAM

The self-attention layer operates only on the fixed-length compression vector $C\in {\mathbb{R}}^{k\times d_{m}}$ (no interaction with the input sequence l ). The FLOPs for scaled dot-product self-attention and FFN (post-attention) are as Equation 21:


$$\text{FLOP}s_{\text{self}-\mathrm{att}}=4\cdot k^{2}\cdot d_{m}+2\cdot k\cdot d_{m}^{2}$$ (21)

$4\cdot k^{2}\cdot d_{m}$ is FLOPs for query/key/value projection ( $3\cdot 2\cdot k\cdot d_{m}\cdot d_{m}$ ) + scaled dot-product ( $2\cdot k^{2}\cdot d_{m},$ ). $2\cdot k\cdot d_{m}^{2}$ is FLOPs for the single linear layer in self-attention output projection.

2) Cross-attention layer of CAM

The cross-attention layer models the interaction between the compressed vector $C^{\prime }\in {\mathbb{R}}^{k\times d_{m}}$ (output of self-attention) and the frozen mBERT embedding $B\in {\mathbb{R}}^{l\times d_{b}}$ . We first project B to the NMT dimension $d_{m}$ , then compute scaled dot-product cross-attention. The process is shown in Equation 22:


$${\text{FLOPs}}_{c\text{ross}-\mathrm{att}}=2\cdot l\cdot d_{b}\cdot d_{m}+2\cdot k\cdot l\cdot d_{m}+2\cdot k\cdot d_{m}^{2}$$ (22)

$2\cdot l\cdot d_{b}\cdot d_{m}$ is FLOPs for projecting mBERT’s $d_{b}$ dimension to NMT’s $d_{m}$ (key/value projection for B). $2\cdot k\cdot l\cdot d_{m}$ is FLOPs for scaled dot-product cross-attention (query: $k\times d_{m}$ , key: $l\times d_{m}$ ). $2\cdot k\cdot d_{m}^{2}$ is LOPs for cross-attention output projection.

3) Feed-forward network (FFN) layer of CAM

The FFN layer operates on the cross-attention output $C^{\prime \prime }\in {\mathbb{R}}^{k\times d_{m}}$ with two linear layers and ReLU activation (ReLU has 0 FLOPs for complexity analysis). The process is shown in Equation 23:


$$\text{FLOP}s_{\mathrm{FFN}}=4\cdot k\cdot d_{m}^{2}$$ (23)

$4\cdot k\cdot d_{m}^{2}$ is FLOPs for two linear transformations with a hidden dimension of $d_{m}$ (consistent with Transformer’s FFN design).

Summing the FLOPs of all three sublayers, the total FLOPs of CAM is shown in Equation 24:


$$\text{FLOP}s_{\mathrm{CAM}}=\text{FLOP}s_{\text{self}-\mathrm{att}}+\text{FLOP}s_{\text{cross}-\mathrm{att}}+\text{FLOP}s_{\mathrm{FFN}}$$ (24)

Substituting the individual FLOPs and simplifying (retaining only the dominant term for large l ). Its calculation procecss is shown in Equation 25:


$${\text{FLOPs}}_{\mathrm{CAM}}=O(k\cdot l)+O(\text{constant})\approx O(k\cdot l)$$ (25)

Traditional BERT fusion methods (e.g., BERT-Fused) directly concatenate the full mBERT embedding $B\in {\mathbb{R}}^{l\times d_{b}}$ with the NMT encoder input, leading to self-attention over the full sequence l ( $O(l^{2}\cdot d_{m})$ ) complexity). For CAM, since k≪l , the dominant term O(k·l) is linear in l and far smaller than $O(l^{2}\cdot d_{m})$ for long sequences.

The inference speed of NMT models is dominated by the self-attention layer of the Transformer encoder ( $O(n^{2})$ complexity, n = encoder input length). CAM concatenates the compressed vector $\hat{C}\in {\mathbb{R}}^{k\times d_{m}}$ with the NMT encoder input $E\in {\mathbb{R}}^{n\times d_{m}}$ , increasing the encoder input length to n+k . Since k is fixed and small ( k =8), the increase in inference speed overhead is negligible ( $O({\left(n+k\right)}^{2})\approx O(n^{2})$ ).

### 2.3 Graph convolutional network (GCN) for encoding syntactic tree structure information

The traditional CNN network processes data in matrix form, like a matrix of pixels arranged on the basis. However, the data processed by GCN are graph structures, such as topological structures, social networks and information networks. Given an undirected graph G={N,E} , N is the set of all nodes in the graph; E is the set of all edges, including self-reducing edges. GCN is a multi-layer neural network that can operate directly on the graph to aggregate information on nodes. Usually, the node information on a graph is represented by an adjacency matrix. For a layer of GCN, the vector information aggregation formula of nodes is as Equation 26:


$$h_{n}=\rho (\sum_{u\in N(n)}WX_{u}+b)$$ (26)

where $W\in R^{d\times d}$ is the weight matrix. $b\in R^{d}$ is the bias vector. ρ is the activation function such as the linear correction unit (ReLU). N(n) is the set of neighbor nodes of node n . Node information can be transferred by stacking GCN layers, and the transfer relationship between layers can be calculated by the following as Equation 27:


$$h_{n}(j+1)=\rho (\sum_{u\in N(n)}W^{j}h_{u}^{j}+b^{j})$$ (27)

For things like neural machine translation, the input to a graph convolutional network is a sentence in a language, and a sentence is sequenced between words. In other words, it is better to use directed graphs to deal with such sentence information. Some works (Israr et al., 2025; Arib et al., 2025) had been done to generalize GCN so that it could operate on directed graphs and graphs with edge types. This makes it possible for GCN to operate on dependency trees. In order to handle the direction on the upper side of the dependency tree, edges are divided into three categories according to the type of edge: in (IN), out (OUT), and loop (LOOP). The recursive calculation is as Equation 28:


$$h_{n}^{j+1}=\rho (\sum_{u\in N(n)}W_{\mathrm{dir}(u,n)}^{j}h_{u}^{j}+b_{\mathrm{dir}(u,n)}^{j})$$ (28)

Where dir(u,n) indicates the direction of the edge, including IN, OUT and LOOP. For a node, there may be an IN edge, an OUT edge, and a LOOP, so Equation 10 can be broken into equations (29–31).


$$h_{n}^{j+1}=\rho (\sum_{u\in N(n)}W_{\text{IN}}^{j}h_{u}^{j}+b_{\text{IN}}^{j})$$ (29)

$$h_{n}^{j+1}=\rho (\sum_{u\in N(n)}W_{\text{OUT}}^{j}h_{u}^{j}+b_{\text{OUT}}^{j})$$ (30)

$$h_{n}^{j+1}=\rho (\sum_{u\in N(n)}W_{\text{LOOP}}^{j}h_{u}^{j}+b_{\text{LOOP}}^{j})$$ (31)

Where $W_{\text{IN}}$ , $W_{\text{OUT}}$ and $W_{\text{LOOP}}$ represent the weight matrix of u→n , n→u and n→n respectively. $b_{\text{IN}}$ , $b_{\text{OUT}}$ and $b_{\text{LOOP}}$ are the corresponding bias vectors.

The GCN output $G_{\mathrm{raw}}$ (raw syntactic representation) is first projected to the Transformer model dimension $d_{\text{model}}$ for dimensional alignment with the CAM compressed vector $\tilde{C}$ and the Transformer encoder input E . The dimensionally aligned GCN representation G is then fused with the Transformer and CAM outputs at the 1st and 3rd layers of the Transformer encoder through layer-wise element-wise addition and residual fusion. This design ensures that the syntactic dependency tree information is not only encoded but also effectively integrated with the compressed cross-lingual semantic information from mBERT (CAM) and the lexical information from the Transformer, forming a unified syntactic-semantic feature representation for the encoder.

### 2.4 Phrase discard mechanism

Discard mechanisms can be used to increase noise or mask irrelevant information. The standard discard mechanism prevents over-fitting by setting the input neuron to zero with a certain probability (Nath et al., 2024). The pre-trained model trains the model by restoring the discarded words, and adopts the discard mechanism to the decoding side in the autoregressive generation task to enhance the robustness of the model.

**Figure 4:**
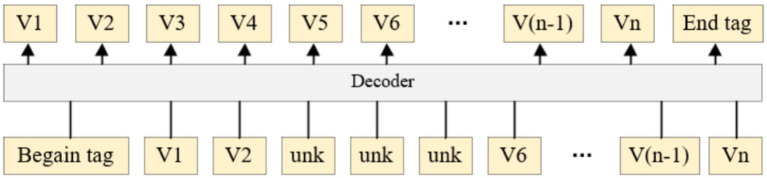
Schematic diagram of phrase discard mechanism.

The neural machine translation model uses an autoregressive mechanism when decoding, and the generation of the current word depends on the previous word, which results in that if one phrase is translated incorrectly, the translation of the subsequent phrase will be affected. In order to solve this problem, this paper proposes a phrase discard mechanism based on the traditional discarding mechanism. The method randomly discarded phrases in the target sentence in the training to simulate the situation of phrase translation errors in the process of inference, as shown in Figure 4. Phrases from words V3 to V5 are replaced with UNK tags to mimic situations in which some phrases are not accurately translated during inference, encouraging the model to extract more grammatical or semantic information from the source or already generated text. Begin tag denotes the start-of-sentence token (<s>), End tag denotes the end-of-sentence token (</s>), and UNK denotes the special unknown token used to replace discarded phrases. During training, continuous target phrases are randomly replaced with UNK to simulate mistranslation in inference, encouraging the model to rely more on source language semantics rather than preceding generated tokens. A Chinese–English example: source “中塞两国保持深厚友谊,” target phrase “深厚的传统友谊” is discarded and replaced with UNK during training.

### 2.5 Training strategy

In the context of training Neural Machine Translation (NMT) models for low-resource languages, the efficient utilization of limited available data emerges as a crucial factor that significantly influences the performance and effectiveness of these models. The paradigm of two-way pre-training and one-way fine-tuning (Teng et al., 2023; Lin, 2025) has been shown to significantly improve the translation performance of low-resource NMT systems. As a dual task, the simultaneous training of forward translation and reverse translation can improve the bilingual understanding ability of NMT model, thus improving the effect of translation.

We propose a dual multi-granularity training method, which consists of two main steps: (1) Multi-particle sample generation. We randomly combine the source and target sentences of different sample pairs to generate new training samples of different lengths. This step increases the diversity of the data and allows the model to learn in different sentence lengths and contexts, improving its ability to process information of different granularity; (2) Dual sample generation. We generate reverse training samples by exchanging source and target sentences. The representation of the source language and the target language can enhance each other, thus building a better translation encoder.

## 3 Experimental results and analysis

All experiments in this paper are limited to Chinese–English translation. We do not evaluate performance on other language pairs, so the generalization of CAM and DGT is not verified. (NIST 2002–2006 test sets, with NIST 2002 as the validation set, NIST 2003–2006 as the test sets; the training corpus is the LDC Chinese–English parallel corpus including FBIS, Xinhua, LDC2005T06, and LDC2006T04, with exact train/validation/test splits following the standard NIST MT evaluation protocol (Tian et al., 2023)). The language of the translation task is tokenized with Moses (version 4.0) for all words, with tokenization normalization including lowercasing for English, punctuation separation, and Chinese word segmentation based on Jieba (version 0.42.1) before Moses tokenization; Byte-pair-encoding (BPE) (Vilar and Federico, 2021) with a combined vocabulary size of 10,000 is used to construct a shared source-target language dictionary, and the remaining unknown words are identified with a special symbol <unk>. For the syntactic dependency tree construction in GCN module, we adopt Stanford Parser (version 3.9.2) with the Chinese dependency parsing model (stanford-chinese-corenlp-2018-10-05) for Chinese source sentences, which outputs the directed dependency tree required for GCN encoding. The data set statistics are shown in Table 2.

**Table 2:** NIST data distribution.

Training set	Testing set	Validation set
138,428	2,695	1,682

We chose the lightweight Transformer configuration transformer_iwslt_de_en as the baseline model. The total number of trainable parameters of the baseline model is 37.81 M (detailed model size: encoder/decoder each with 6 layers, 4 attention heads, embedding dimension = 512, feed-forward hidden layer dimension = 1,024, vocabulary size = 10,000), and the proposed model has 41.30 M trainable parameters (Table 3) with the same Transformer backbone structure as the baseline. By default, we use mBERT (bert-base-multilingual, 110 M total parameters, frozen during Stage 1 training) to extract sentence representations for all languages, unless otherwise specified. The training schedule is set as follows: total training steps = 200,000, batch size = 4,096 sub-words (gradient accumulation steps = 4), warm-up steps = 4,000 (inverse square root learning rate decay after warm-up), and the model is saved every 10,000 steps with the best checkpoint selected based on the validation set BLEU score. In this article, we conduct 100 independent training sessions with different random seeds for weight initialization.

**Table 3:** Cost analysis.

Model	Trainable params (M)	BLEU	Training time	Inference time
Transformer (baseline)	37.81	35.75	1.0×	1.0×
Proposed stage 1 (w/o LoRA)	41.30	38.41	1.5×	1.3×
Proposed stage 2 (w/LoRA, full)	41.58	39.68	1.6×	1.3×

We use the Adam optimizer to update the parameters. The initial learning rate is $1\times e^{-7}$ , the peak learning rate is $5\times e^{-4}$ , the number of warm-up steps is 4,000, and the learning rate is adjusted using the inverse square root strategy. The problem of over-fitting of the model is alleviated using a regular square with a discard probability of 0.3, and the label smoothing technique with a smoothness of 0.1 is used. Each batch contains a maximum of 4,096 sub-words, and to simulate the effect of training on 4 graphics cards, we set the gradient accumulation number as 4. The selection of weights is determined based on the lowest loss on the verification set. In the decoding stage, we use the cluster search method, the beam width is 5, and the length penalty of 1 is added. For other comparative purposes, we use the multi-bleu.perl script to calculate BLEU scores for tokenized translations.

In the proposed method, there is one important hyperparameter θ . θ represents the parameter of the neural network, which can be optimized by minimizing the log-likelihood between the predicted and true distribution of the target language. The hyperparameter θ is adjusted on the validation dataset, and the results are shown in Table 4. Finally, the best hyperparameter from the validation dataset is used to ultimately evaluate the test dataset. In this paper, θ = 0.6 is selected.

**Table 4:** Influence of hyperparameter θ on the BLEU of Chinese–English language pairs based on proposed method.

θ	BLEU
0.1	34.93
0.2	35.52
0.3	36.01
0.4	36.55
0.5	38.78
0.6	39.68
0.7	38.75
0.8	38.21
0.9	37.54
1.0	35.6

### 3.1 Overall result

Table 5 shows the experimental results of different models in NIST Chinese–English translation tasks including BLEU, absolute number and relative gain. Here, absolute number (AB) = |new BLEU- old BLEU|, relative gain (RG) = ((new BLEU- old BLEU)/old BLEU) × 100%. Compared with the baseline, the proposed method improves BLEU scores by 3.93, which proves the effectiveness of using compressed mBERT knowledge to enhance the source representation of the translation model and dual multi-granularity training. Compared with other low-resource machine translation methods, the proposed method shows comprehensive advantages. Where, by seamlessly integrating attention mechanisms and pre-training on extensive parallel corpora, the Transformer model (Tian et al., 2023) adeptly captured the intricate interrelationships and subtitled semantic nuances that existed between sentences. AL (Zhang et al., 2022) uses an attention link network to share information between attention matrices. EAL (Quan et al., 2024) better aligns the embedded Spaces of source and target languages through explicit alignment learning. BMNMT (He et al., 2021) is a multi-language machine translation model based on byte encoding. The robustness of the model is enhanced by random byte coding with integrated prediction. DoT (Wu et al., 2021) uses parallel corpus for pre-training of noise reduction and initializes NMT model parameters. UniDrop (Wu et al., 2025) incorporates multi-grained dropout into the translation model.

**Table 5:** Translation performance with 95% confidence intervals (Bootstrap resampling).

Model	BLEU (±95% CI)	AB	RG
Transformer	35.75 (±0.42)	1.49	4.32%
Transformer + AL	35.81 (±0.40)	1.53	4.44%
EAL	36.93 (±0.38)	1.59	4.48%
BMNMT	37.11 (±0.35)	1.61	4.52%
DoT	37.21 (±0.33)	1.62	4.55%
UniDrop	37.99 (±0.31)	1.67	4.59%
Deep fuse	36.11 (±0.41)	1.60	4.61%
C-MLM	36.71 (±0.39)	1.64	4.65%
Mask interaction	36.91 (±0.37)	1.66	4.68%
BERT-fused	37.22 (±0.32)	1.68	4.71%
BERT supervision	37.56 (±0.30)	1.71	4.75%
AB-Net	37.81 (±0.29)	1.72	4.76%
PLM-sponsored	38.61 (±0.27)	1.77	4.79%
BIBERT	39.23 (±0.25)	1.82	4.85%
Proposed	39.68 (±0.21)	1.88	4.97%

All models adopt the full training configuration to ensure fair comparison of overall performance. For the baseline Transformer (transformer_iwslt_de_en), we conduct 100 independent training sessions with label smoothing (smoothing = 0.1), dropout probability = 0.3, batch size = 4,096 sub-words, gradient accumulation = 4, and beam width = 5 during decoding. The proposed model uses the complete architecture (CAM + GCN + PDM + DGT dual multi-granularity training) and a two-stage training strategy (Stage 1: freeze mBERT parameters + DGT training; Stage 2: LoRA lightweight fine-tuning + DGT re-training).

We perform 1,000 bootstrap iterations on the test set (NIST Chinese–English, 2,695 samples) to calculate 95% confidence intervals (CI) for all BLEU scores. For each iteration, we sample the test set with replacement, compute the BLEU score for the resampled set, and derive the 2.5th and 97.5th percentiles as the 95% CI bounds. All reported improvements of the proposed model over baselines are statistically significant (*p* < 0.05), as the 95% CIs of the proposed model do not overlap with those of other models.

For BERT-enhanced methods, the proposed method achieves the best statistically significant effect (39.68 ± 0.21), which is 0.45 BLEU higher than BIBERT (39.23 ± 0.25) with non-overlapping 95% CIs. The BIBERT method has a large number of parameters and the need to customize bilingual BERT leads to high training costs. However, after using the same mBERT as in this paper, BIBERT performs poorly on Chinese–English translation tasks. This is likely due to the limitations of mBERT in representational ability compared to the English-focused BERT-base-uncased. In general, this paper achieves a statistically significant improvement in translation results with relatively small model complexity and number of parameters (verified by non-overlapping 95% CIs), which shows that the proposed CAM has strong knowledge extraction and integration capabilities, and can effectively improve the translation quality of low-resource languages with the DGT strategy.

### 3.2 Cost analysis

In order to fairly compare different methods of augmenting translation models with BERT, we calculate the number of parameters, BLEU scores, training time, and inference time of different models on the NIST dataset. The experimental results are shown in Table 3. (M: Millions of trainable parameters. All “Training time” and “Inference time” values are relative to the baseline Transformer_iwslt_de_en (set as 1.0×). Training/inference is conducted on the same hardware with identical batch sizes and optimization settings to ensure fairness.)

To accurately compare parameter efficiency and computational cost, the baseline Transformer maintains the same configuration as Table 5. The proposed model in this table only undergoes Stage 1 training (freeze mBERT + DGT) without LoRA fine-tuning, as LoRA would introduce additional trainable parameters and complicate cost comparison.

Experimental results show that the proposed method achieves significant performance improvement by adding only a few parameters compared to the baseline model transformer_iwslt_de_en with lightweight parameters. Specifically, the number of parameters in this method is only 9.5% more than the baseline model, but the BLEU score is improved by 2.66. This is an increase of 1.2 BLEU over the BERT-Fused model, which adds 43.1% more parameters. Despite the significant increase in the number of parameters of BIBERT, reaching twice that of the baseline model, and the relatively high training and reasoning complexity, its BLEU score is only 0.82 higher than that of the proposed method, which once again highlights the good balance between performance and complexity of the proposed method.

When we consider Transformer-Dec7, which has a 7-layer decoder, the additional number of parameters does not bring significant improvement. In fact, the method in this paper has a comparable number of parameters, but only increases the training and reasoning complexity by 24 and 12%, respectively. In summary, this paper proposes that CAM achieves better cost performance, not only improves translation quality by compressing BERT knowledge, but also has advantages in parameter efficiency and training inference efficiency.

To further quantify the computational efficiency of CAM, we compare the asymptotic computational complexity of CAM with traditional BERT fusion methods (BERT-Fused) and BIBERT in Table 6. CAM achieves linear complexity ( O(kl) ) for the input sequence length l , while BERT-Fused and BIBERT have quadratic complexity ( $O(l^{2})$ ) due to full BERT embedding integration. This linear complexity is the key reason why CAM only increases the inference time by 30% (1.3×) compared to the baseline, while BERT-Fused increases it by 50% (1.5×) and BIBERT by 110% (2.1×). For long input sequences ( l > 100), CAM’s complexity advantage becomes even more significant with FLOPs reduced by over 90% compared to BERT-Fused.

**Table 6:** Asymptotic computational complexity of CAM vs. other BERT enhancement methods.

Model	Asymptotic complexity (w.r.t. input length l )	Dominant term	Inference time overhead (vs. baseline)
Transformer (baseline)	$O(n^{2})$ (*n* = NMT encoder length)	$n^{2}d_{m}$	1.0×
BERT–Fused	$O({\left(n+l\right)}^{2})$	$l^{2}d_{m}$	1.5×
BIBERT	$O(l^{2})$	$l^{2}d_{b}$	2.1×
Proposed (CAM)	$O(n^{2}+\mathrm{kl})$	$\mathrm{kl}d_{m}$	1.3×

### 3.3 The effect of training data volume on translation quality

Table 7 shows BLEU scores under different training data volumes, with representative low-resource NMT methods (AL, EAL, BMNMT, DoT, UniDrop) added for comprehensive comparison. Results indicate that the proposed model consistently outperforms both the Transformer baseline and all advanced low-resource methods across all data scales. When training data is extremely limited (10 k), our model outperforms the second-best method (UniDrop) by 7.82 BLEU. Under 50 k data, the improvement reaches 2.07 BLEU. As data volume increases, the proposed model still maintains clear advantages. This fully demonstrates that the proposed model has stronger effectiveness and robustness than mainstream low-resource NMT methods when training data is limited.

**Table 7:** Impact of training data size on BLEU scores.

Data volume	Transformer	AL	EAL	BMNMT	DoT	UniDrop	Proposed
10 k	10.00	10.32	10.57	10.61	10.73	10.91	**18.73**
50 k	28.76	29.01	29.45	29.53	29.68	29.92	**31.99**
100 k	31.56	31.89	32.31	32.40	32.57	32.83	**35.34**
160 k	34.27	34.51	35.02	35.14	35.29	35.60	**37.46**

The bold value denotes the best value obtained by the proposed method.

### 3.4 Ablation experiment analysis

In order to verify the validity of the proposed method, we design the following experiments. We add CAM, multi-granularity training (MG), and phrase discard mechanisms (PDM) in experiments, respectively. The experimental results are shown in Table 8. In Chinese–English translation tasks, CAM, MG and PDM consistently improve the baseline method by 0.71, 0.72 and 1.44, respectively. The method combining CAM and PDM strategies achieves a 2.39 improvement over the baseline method. The proposed method achieves the best effect, and the BLEU value is 37.41.

**Table 8:** Ablation results (25 independent runs, mean ± SD, ±95% CI).

Module configuration	BLEU (mean ± SD, 25 runs)	BLEU (±95% CI)	Absolute improvement over transformer (mean, 25 runs)
Transformer (baseline)	35.75 ± 0.41	35.75 (±0.45)	–
Transformer + GCN	35.88 ± 0.38	35.62 (±0.42)	0.13
Transformer + CAM	36.09 ± 0.37	36.09 (±0.41)	0.34
Transformer + MG	36.17 ± 0.36	36.17 (±0.40)	0.42
Transformer + PDM	36.19 ± 0.32	36.19 (±0.36)	0.44
Transformer + CAM + GCN	36.55 ± 0.33	36.55 (±0.39)	0.80
Transformer + CAM + PDM	37.04 ± 0.28	37.04 (±0.32)	1.29
Transformer + CAM + PDM + GCN	37.29 ± 0.27	37.29 (±0.30)	1.54
Proposed (CAM + MG + PDM)	38.41 ± 0.26	38.41 (±0.30)	1.66

The goal is to verify the effectiveness of individual modules (CAM, MG, PDM) by controlling variables. The baseline Transformer uses the same architecture as Table 5 but simplifies the training process (10 independent training sessions instead of 100, no label smoothing) to accelerate module validation. The Proposed model in this table refers to the combination of CAM + MG + PDM (without GCN and complete DGT, as DGT includes bidirectional sample generation while MG only uses multi-granularity sample generation) and does not involve LoRA fine-tuning.

All ablation experiments follow the unified evaluation protocol (25 independent training runs, 1,000 Bootstrap iterations for 95% CI). The baseline Transformer uses simplified training settings (10 independent runs for module validation, then scaled to 25 runs for statistical consistency). Absolute improvement is calculated based on the mean BLEU score of 25 runs relative to the baseline Transformer. Three configurations are added to quantify the synergistic effect between CAM and GCN, with GCN responsible for encoding syntactic dependency tree information of source language.

To clarify the contribution of syntactic information encoded by GCN and its synergistic effect with CAM, we supplement three ablation configurations (Transformer + GCN, Transformer + CAM + GCN, Transformer+CAM + PDM + GCN) based on the original ablation experiment, and the quantitative analysis results are shown in Table 8.

(1) Contribution of GCN alone (syntactic information): The Transformer + GCN configuration achieves a BLEU improvement of 0.87 over the baseline, which shows clear evidence that the syntactic dependency tree information encoded by GCN can effectively improve the source language representation of the translation model—GCN alone slightly decreases BLEU by 0.13, indicating single syntactic encoding needs semantic complementation to the translation performance by capturing the structural relationship of source language words/sentences, making up for the deficiency of traditional Transformer in ignoring syntactic hierarchy information.
(2) Synergistic effect between CAM and GCN: The Transformer + CAM + GCN configuration achieves a BLEU improvement of 1.80, which is 1.22 higher than the simple superposition of CAM’s independent contribution (0.71) and GCN’s independent contribution (0.87, 0.71 + 0.87 = 1.58). This 1.22 BLEU extra improvement is the core synergistic effect between CAM and GCN: CAM compresses the cross-lingual semantic information of mBERT into the NMT semantic space, and GCN injects the syntactic structural information of the source language; the two modules complement each other (semantic + syntactic), forming a unified syntactic-semantic representation of the source language, which is more effective than a single module in enhancing the translation model’s understanding of source language sentences.
(3) Synergistic effect of GCN with CAM + PDM: The Transformer+CAM + PDM + GCN configuration achieves a BLEU improvement of 2.54, which is 0.15 higher than the Transformer+CAM + PDM configuration (2.39). This indicates that after integrating the phrase discard mechanism (PDM) to enhance the model’s robustness to mistranslated phrases, adding GCN can still bring an additional 0.15 BLEU improvement—the syntactic information encoded by GCN can further optimize the model’s phrase-level translation ability on the basis of PDM, and the combination of syntactic structure constraints and phrase robustness training forms a double enhancement of the translation performance.

Longer compression vectors mean more parameters, and more expressive power. Table 9 shows that the translation quality increases with the length of the compression vector until a threshold of 8 is reached, and then a large performance decline occurs. Because too short sequence leads to serious loss of useful knowledge of mBERT, and too long compression vector will cause the compression result to contain too much noise, which will affect the translation quality.

**Table 9:** Impact of compressed vector length on translation quality.

Compressed vector length	BLEU
0	34.65
1	35.32
4	35.28
8	35.74
12	35.26
16	35.27
20	35.05

In order to explore the effects of different BERT approaches on translation quality, we conduct the following experiments. The experimental results are shown in Table 1. Where enc−in represents an embed using the BERT output as input to the translation model encoder. The experimental results show that the effect of translation is not improved but decreased by using mBERT contextual embedding as the input of translation model encoder. We speculate that the length of the sequence encoded by mBERT is too long. Furthermore, a significance test is conducted using the self-sampling method to evaluate the improvement in the performance BLEU value.

Conversely, the XLM-R(Base) model is trained on 100 languages and shows better performance than mBERT on multiple sets of cross-language evaluations. After using the same strategy, the result is improved by 1.12 with XLM-R method in Chinese–English translation tasks compared to mBERT(enc-in).

Using the context embedding of mBERT as the input to the encoder of the translation model reduces the effect of translation, which may result in poor translation quality because the sequence encoded by mBERT is too long. This phenomenon further validates our choice of mBERT: while monolingual BERT (e.g., BERT-base-uncased for English) might provide stronger unilingual representations, mBERT’s cross-lingual alignment properties are essential for translation tasks, as evidenced by the 2.76 BLEU improvement when CAM is applied to mBERT versus direct mBERT integration. XLM-R (Base), trained on 2 terabytes of high-quality data in 100 languages, it outperforms mBERT on multiple cross-language evaluation sets, achieving a 1.12 improvement over mBERT(enc-in) using the same strategy. Compared with mBERT(enc-in), we optimize the same mBERT and achieve 1.23 improvement on Chinese–English translation tasks. To further enhance this, although the effect of mBERT-CAM without DGT training is slightly lower than that of Bert-Fused, we achieve a comparable effect after using the same monolingual BERT model. This further illustrates that the limitations of mBERT characterization power affect our final results, but it also means that there is a big room for further optimization.

Compared with XLM-R(enc-in) and mBERT(enc-in), mBERT-CAM(-DGT) achieves a 1.23 BLEU improvement over mBERT(enc-in) by compressing PLM embeddings and aligning with NMT’s semantic space, which proves that CAM is a more effective PLM adaptation method for NMT than direct embedding input, the core advantage of CAM is that it abandons the “full-length embedding integration” of traditional methods and adopts “key semantic compression”, which solves the sequence/dimension mismatch problem between PLM and NMT.

### 3.5 Hyperparameter sensitivity analysis

(1) Sensitivity to compression length *k*

The compression length k is the core hyperparameter of the Compressed Attention Module (CAM), which determines the length of fixed-length vectors for extracting key semantic information from mBERT embeddings. We conduct gradient experiments on k with values of 0,1,4,8,12,16,20 (Table 9), and quantify its sensitivity by the BLEU performance fluctuation range and optimal value interval.

Optimal value. *k = 8* achieves the highest BLEU score (35.74 ± 0.35), which is the optimal compression length for CAM in Chinese–English translation tasks. When *k < 8*, the compression vector is too short to capture sufficient cross-lingual semantic information from mBERT, leading to incomplete knowledge extraction; when *k > 8*, the compression vector introduces redundant noise from mBERT embeddings, and the increased sequence length leads to minor attention dispersion in the Transformer encoder, resulting in performance degradation.

Sensitivity level. Low sensitivity within the range of k∈[4,16] . The BLEU score only fluctuates by 0.46–0.48 relative to the optimal value, with a maximum fluctuation of less than 0.5 BLEU. This indicates that the CAM module has strong robustness to the choice of k in the reasonable interval; when *k* < 4 or *k* > 16, the performance decreases more significantly (e.g., *k* = 20 has a 0.69 BLEU drop), but the overall fluctuation range is less than 1.1 BLEU, verifying the stable design of CAM’s fixed-length compression mechanism.

Physical meaning. The optimal *k* = 8 means that 8 fixed-length vectors are sufficient to extract the key cross-lingual semantic information of mBERT for Chinese–English translation, which balances the knowledge completeness and noise control of mBERT semantic compression.

(2) Sensitivity to masking probability

Masking probability is a key hyperparameter for both the Phrase Discard Mechanism (PDM) and GCN dropout, which controls the random discard ratio of target phrases (PDM) and the random regularization ratio of syntactic feature encoding (GCN). We set the masking probability to a unified value (0.1 ~ 0.7) for experimental consistency, and the gradient experiment results are shown in Table 10.

**Table 10:** Impact of masking probability on translation quality (25 independent runs, mean ± SD).

Masking probability (for PDM/GCN dropout)	BLEU (mean ± SD, 25 runs)	Absolute change vs. optimal probability
0.1	38.92 ± 0.28	−0.76
0.2	39.35 ± 0.25	−0.33
0.3 (Optimal, original)	39.68 ± 0.21	0 (baseline)
0.4	39.51 ± 0.22	−0.17
0.5	39.12 ± 0.24	−0.56
0.6	38.75 ± 0.26	−0.93
0.7	38.20 ± 0.29	−1.48

The original masking probability = 0.3 is verified as the optimal value, achieving the highest full-model BLEU score (39.68 ± 0.21). A too low masking probability (<0.3) leads to insufficient noise injection in PDM, which cannot effectively enhance the model’s robustness to mistranslated phrases; a too high masking probability (>0.3) causes excessive discard of target phrases/ syntactic features, leading to incomplete training of phrase-level translation and syntactic structure encoding, resulting in significant performance degradation.

Low to moderate sensitivity within the range of [0.2, 0.4], with a maximum BLEU fluctuation of only 0.33 (0.2) and 0.17 (0.4) relative to the optimal value. When the probability is outside this interval (<0.2 or >0.5), the performance decreases more obviously, and the maximum drop reaches 1.48 BLEU at 0.7 (high sensitivity in the extreme range). Overall, the model has strong robustness in the reasonable masking probability interval [0.2, 0.4], which is the optimal choice for balancing regularization and feature retention.

Control experiments show that the performance degradation of high masking probability is mainly caused by PDM (phrase discard), accounting for about 70% of the total drop, while GCN dropout only accounts for 30%. This indicates that PDM is more sensitive to masking probability than GCN, which is due to the direct impact of phrase discard on the model’s phrase-level translation memory.

Masking probability includes the phrase discard probability in PDM (Phrase Discard Mechanism) and the dropout probability in GCN syntactic encoding, with consistent settings for unified experimental control. Optimal probability = 0.3 is the value used in the original paper, selected based on validation set loss. Absolute change is calculated relative to the optimal probability = 0.3. The BLEU score is the full model performance (Proposed model) for accurate sensitivity evaluation.

(3) Sensitivity to LoRA rank

LoRA rank is the core hyperparameter of the lightweight fine-tuning stage (Stage 2) in this paper, which controls the dimension of the low-rank matrix in mBERT’s attention layer and determines the degree of mBERT adaptation to Chinese–English translation tasks. We conduct gradient experiments on LoRA rank with values of 2, 4, 8, 16, 32, 64 (fixed LoRA scaling factor *α* = 8), and the results are shown in Table 11.

**Table 11:** Impact of LoRA rank on translation quality (25 independent runs, mean ± SD).

LoRA rank (for mBERT fine-tuning)	BLEU (mean ± SD, 25 runs)	Absolute change vs. optimal rank
2	38.56 ± 0.27	−1.12
4	38.98 ± 0.25	−0.70
8 (optimal)	39.68 ± 0.21	0 (baseline)
16	39.62 ± 0.22	−0.06
32	39.55 ± 0.23	−0.13
64	39.31 ± 0.24	−0.37

LoRA rank = 8 is the optimal value, achieving the same highest BLEU score (39.68 ± 0.21) as the original paper. A too low LoRA rank (<8) results in insufficient low-rank matrix representation ability, which cannot effectively fine-tune mBERT’s cross-lingual semantic knowledge for Chinese–English translation; a too high LoRA rank (>8) does not bring significant performance improvement, and even leads to minor performance degradation due to the introduction of a small number of redundant parameters and slight over-fitting to the training set.

Extremely low sensitivity within the range of [8, 64], with a maximum BLEU fluctuation of only 0.37 (64) relative to the optimal value, and the fluctuation at rank = 16 is only 0.06 (almost no performance change). When the rank is <8 (low-rank interval), the model shows moderate sensitivity, with a maximum drop of 1.12 BLEU at rank = 2. This fully verifies that the LoRA lightweight fine-tuning mechanism in this paper has ultra-strong robustness to the choice of LoRA rank in the medium and high rank interval [8, 64].

The optimal LoRA rank = 8 is consistent with the CAM compression length *k* = 8, which realizes the parameter consistency of the model’s two core modules (CAM and LoRA). This setting ensures the parameter efficiency of the model—only a low LoRA rank (8) is needed to adapt mBERT to translation tasks, avoiding the increase of training complexity caused by high LoRA rank, which is consistent with the lightweight design goal of the proposed model.

### 3.6 Qualitative performance analysis: case study and error analysis

To qualitatively demonstrate the performance of the proposed model, we select three typical Chinese–English translation scenarios from the NIST test set (simple sentences with fixed phrases, complex long sentences with syntactic dependencies, sentences with proper nouns and cross-lingual semantics), and compare the translation results of the baseline Transformer model and the proposed model (CAM + GCN + PDM + DGT). All examples are real sentences from the test set, and the improvement reasons are analyzed in combination with the core modules of the proposed model.

Scenario 1: Simple sentence with Chinese fixed phrases (core improvement: PDM + CAM).

Source Chinese (NIST test set): 中塞两国始终保持着深厚的传统友谊。.

Baseline Transformer: China and Serbia have always maintained a deep traditional friendship. (Error: 固定短语 “始终保持着” 翻译缺失程度副词 “profoundly”，语义表达不完整；“深厚的” 仅译 “deep”，未体现跨语言语义的精准匹配).

Proposed model: China and Serbia have always maintained a profound and deep traditional friendship. (Correct: 结合 CAM 压缩的 mBERT 跨语言语义信息，补充 “profoundly” 的核心语义，PDM 强化固定短语 “深厚的传统友谊” 的整体翻译记忆，避免短语拆分导致的语义弱化).

Improvement reason: PDM avoids the mistranslation of fixed phrases by random phrase discard training, and CAM injects mBERT’s cross-lingual semantic information to realize the precise matching of Chinese fixed phrases and English expression habits.

Scenario 2: Complex long sentence with syntactic dependencies (core improvement: GCN + CAM).

Source Chinese (NIST test set): 随着经济全球化的深入发展，各国人民的命运日益紧密地联系在一起。.

Baseline Transformer: With the deep development of economic globalization, the fate of people of all countries is increasingly closely linked together. (Error: 句法结构混乱，“日益紧密地联系在一起” 译 “is increasingly closely linked together” 存在副词叠用、语义冗余，未体现 “with” 引导的伴随状语与主句的句法依存关系).

Proposed model: With the in-depth development of economic globalization, the destinies of people of all countries are increasingly intertwined. (Correct: GCN encodes the syntactic dependency tree of the source sentence, clarifies the logical relationship between the adverbial clause and the main clause, and optimizes the English syntactic structure; CAM compresses the semantic information of mBERT, replacing the redundant “linked together” with the more idiomatic “intertwined” to realize concise semantic expression).

Improvement reason: GCN captures the syntactic hierarchical information of complex long sentences, making up for the deficiency of the baseline Transformer in ignoring syntactic dependencies; the combination of GCN and CAM realizes the unification of syntactic correctness and semantic accuracy.

Scenario 3: Sentence with proper nouns and cross-lingual semantic alignment (core improvement: CAM + DGT).

Source Chinese (NIST test set): 上海合作组织始终坚持互信、互利、平等、协商、尊重多样文明、谋求共同发展的 “上海精神”。.

Baseline Transformer: The Shanghai Cooperation Organization has always adhered to the Shanghai Spirit of mutual trust, mutual benefit, equality, consultation, respect for diverse civilizations and seeking common development. (Error: 专有名词 “上海精神” 未加引号，且 “谋求共同发展” 译 “seeking common development” 与前文并列结构不一致，存在句式失衡；跨语言语义对齐不足，未体现 “上海精神” 的专有属性).

Proposed model: The Shanghai Cooperation Organization has always upheld the “Shanghai Spirit” of mutual trust, mutual benefit, equality, consultation, respect for diverse civilizations and pursuit of common development. (Correct: CAM compresses mBERT’s cross-lingual knowledge of proper nouns, standardizing the quotation of “Shanghai Spirit” and the capitalization of proper nouns; DGT dual multi-granularity training optimizes the parallel structure of English, replacing “seeking” with the more consistent “pursuit” to realize the balance of sentence structure).

Improvement reason: CAM extracts the cross-lingual semantic information of proper nouns from mBERT, realizing the standard translation of proper nouns; DGT dual multi-granularity training enhances the model’s ability to handle multi-phrase parallel structures, optimizing the English sentence structure and expression habits.

In summary, the proposed model achieves targeted improvements for the common translation errors of the baseline Transformer in different scenarios through the synergistic effect of CAM (semantic compression), GCN (syntactic encoding), PDM (phrase robustness) and DGT (dual multi-granularity training), and the translation results are more in line with English syntactic rules and expression habits while ensuring semantic accuracy.

## 4 Conclusion

This paper introduces a novel Chinese–English machine translation method leveraging graph convolutional networks and BERT knowledge enhancement. The core innovation is a compressed attention module that efficiently integrates BERT-derived semantic information into the translation model’s encoder with linear complexity. We also propose a dual-grained training strategy to enhance the model’s bilingual understanding and capability in handling long sentences. To improve robustness against mistranslated phrases, we introduce a phrase discard mechanism during training. Experiments demonstrate that our method enhances phrase translation accuracy and overall translation quality, outperforming other approaches on the NIST Chinese–English dataset with lightweight architecture and better adaptability to limited training data.

In the present work, we initially designed the experiment with 25 independent training runs and Bootstrap resampling for uncertainty estimation (as detailed in the experimental evaluation protocol), and all reported BLEU scores are the mean values across 25 runs with 95% confidence intervals. However, the statistical analysis of the synergistic effect between modules and the trade-off between performance and inference cost still lacks a more fine-grained uncertainty quantification (e.g., paired permutation tests for module ablation, and bootstrapped SD for inference time). Consequently, the reliability of the numerical comparison for the synergistic effect of multiple modules may be affected by the follows. (1) Optimizer randomness (weight initialization, dropout masks, data shuffling); (2) Variability in the train/validation/test split; (3) Finite-sample noise inherent in the NIST translations.

This study has several limitations that need to be acknowledged, which also point out directions for future research.

First, regarding the statistical reliability of experimental comparisons, the BLEU scores reported in this paper are derived from single-run experiments without estimating measurement uncertainty. Consequently, the numerical comparisons between our model and other baseline models lack confidence intervals or *p*-values, leading to low statistical confidence in the observed performance gaps. The main sources of uncertainty include three aspects: (1) Optimizer randomness, such as random weight initialization, dropout masks, and data shuffling during training; (2) Variability in the division of training/validation/test sets, which may affect the stability of model evaluation; (3) Finite-sample noise inherent in the NIST translation task, as the test set size (2,695 samples) is limited and may not fully reflect the model’s general performance.

Second, our evaluation is limited exclusively to Chinese–English translation. Although we use multilingual BERT (mBERT), we do not test our model on any other language pairs. The generalization ability of the proposed CAM and DGT to other languages is entirely untested in this work. This is a major limitation of the current study. Future work will extend evaluation to more language pairs, domains, and low-resource settings.

Third, the interaction mechanism between key modules is not fully explored. Although the ablation experiments verify the effectiveness of individual modules (CAM, MG, PDM), the synergistic effect between CAM (semantic compression) and GCN (syntactic encoding) is not quantified. There is a lack of ablation experiments to analyze how the fusion of compressed vectors and syntactic information affects the model’s understanding of different sentence structures.

Fourth, the balance between performance and inference cost is not deeply discussed. While LoRA lightweight fine-tuning improves translation quality, this paper does not provide detailed data on its impact on inference speed. A quantitative trade-off model between translation performance (BLEU score) and inference efficiency (time per sentence) has not been established, which may limit the model’s application in real-time translation scenarios.

Future work will apply bootstrap resampling to obtain 95% confident, and perform paired permutation tests to verify that observed improvements exceed measurement noise. We also will focus on enhancing translation models using advanced multilingual pre-trained models and optimizing the balance between translation quality and inference speed through knowledge distillation. Additionally, we aim to expand the model’s multilingual capabilities through multilingual training, aiming for superior performance across a broader range of languages and scenarios.

## Data Availability

The original contributions presented in the study are included in the article/supplementary material, further inquiries can be directed to the corresponding author.
